# Acute performance and physiological responses to repeated‐sprint exercise in a combined hot and hypoxic environment

**DOI:** 10.14814/phy2.14466

**Published:** 2020-06-26

**Authors:** Keiichi Yamaguchi, Nobukazu Kasai, Nanako Hayashi, Haruka Yatsutani, Olivier Girard, Kazushige Goto

**Affiliations:** ^1^ Graduate School of Sport and Health Science Ritsumeikan University Kusatsu Shiga Japan; ^2^ Department of Sports Science Japan Institute of Sports Sciences Kitaku Tokyo Japan; ^3^ School of Human Science (Exercise and Sport Science) The University of Western Australia Crawley Australia

**Keywords:** combination of stressors, heat stress, hypoxia, repeated‐sprints

## Abstract

We investigated performance, energy metabolism, acid–base balance, and endocrine responses to repeated‐sprint exercise in hot and/or hypoxic environment. In a single‐blind, cross‐over study, 10 male highly trained athletes completed a repeated cycle sprint exercise (3 sets of 3 × 10‐s maximal sprints with 40‐s passive recovery) under four conditions (control [CON; 20℃, 50% rH, FiO_2_: 20.9%; sea level], hypoxia [HYP; 20℃, 50% rH, FiO_2_: 14.5%; a simulated altitude of 3,000 m], hot [HOT; 35℃, 50% rH, FiO_2_: 20.9%; sea level], and hot + hypoxia [HH; 35℃, 50% rH, FiO_2_: 14.5%; a simulated altitude of 3,000 m]). Changes in power output, muscle and skin temperatures, and respiratory oxygen uptake were measured. Peak (CON: 912 ± 26 W, 95% confidence interval [CI]: 862–962 W, HYP: 915 ± 28 W [CI: 860–970 W], HOT: 937 ± 26 W [CI: 887–987 W], HH: 937 ± 26 W [CI: 886–987 W]) and mean (CON: 808 ± 22 W [CI: 765–851 W], HYP: 810 ± 23 W [CI: 765–855 W], HOT: 825 ± 22 W [CI: 781–868 W], HH: 824 ± 25 W [CI: 776–873 W]) power outputs were significantly greater when exercising in heat conditions (HOT and HH) during the first sprint (*p* < .05). Heat exposure (HOT and HH) elevated muscle and skin temperatures compared to other conditions (*p* < .05). Oxygen uptake and arterial oxygen saturation were significantly lower in hypoxic conditions (HYP and HH) versus the other conditions (*p* < .05). In summary, additional heat stress when sprinting repeatedly in hypoxia improved performance (early during exercise), while maintaining low arterial oxygen saturation.

## Graphical abstract

The present study investigated performance, energy metabolism, acid‐base balance, and endocrine responses to repeated sprint exercise in a hot and/or hypoxic environment. Our findings suggest that additional heat stress to hypoxia enhances repeated sprint performance with maintaining low arterial oxygen saturation.

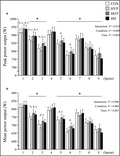

## 1 INTRODUCTION

Team and racket sport athletes repeatedly produce brief bouts of maximal power output (~10 s) interspersed with insufficient recovery period (~60 s) during competition. This physical fitness component is known as “repeated‐sprint ability” (RSA) (Bishop, Girard, & Mendez‐Villanueva, 2011; Girard, Mendez‐Villanueva, & Bishop, 2011). Recent studies have shown that several weeks of repeated‐sprint training in hypoxia further improves RSA compared with the same training in normoxia (Beard, Ashby, Kilgallon, Brocherie, & Millet, 2019; Brocherie, Girard, Faiss, & Millet, 2017; Kasai et al., 2015). Such larger hypoxia‐induced performance improvements could be mediated by the physiological adaptations that arise from this training, including increased anaerobic glycolysis (Bowtell, Cooke, Turner, Mileva, & Sumners, 2014; Ogura, Katamoto, Uchimaru, Takahashi, & Naito, 2006) and muscle blood perfusion (Faiss et al., 2013).

Although acute hypoxia limits the aerobic energy supply due to reduced systemic oxygen uptake (Ogawa, Hayashi, Ichinose, Wada, & Nishiyasu, 2007; Ogura et al., 2006), it also increases anaerobic glycolysis during high‐intensity exercise (Morales‐Alamo et al., 2012; Weyand et al., 1999). Consequently, the accumulation of metabolites, such as lactate, in working muscles is greater during sprint exercise in hypoxia than in normoxia (Morales‐Alamo et al., 2012). Repeated‐sprint training in hypoxia improves anaerobic glycolytic activity and muscle buffering capacity presumably due to molecular adaptations. For instance, this includes upregulated lactate dehydrogenase activity and mRNA expression of carbonic anhydrase Ⅲ and monocarboxylate transporter 4 (Faiss et al., 2013). Besides, heat stress (whole body exposure in a hot environment or local heating) increases muscle glycogen utilization. Enhanced glycogen utilization during exercise in hot conditions is associated with elevated muscle temperature (Febbraio, Carey, Snow, Stathis, & Hargreaves, 1996; Febbraio, Snow, Stathis, Hargreaves, & Carey, 1994). Altogether, both hypoxia and heat stress activate the glycolytic pathway (glycogen utilization).

Hypoxia does not affect maximal sprint performance during the early stage of a repeated‐sprint exercise. With sprints repetition, however, decrement in power output is exacerbated by limited oxygen (O_2_) availability compared with normoxia, with this effect becoming more visible under more severe hypoxic conditions (e.g., FiO_2_ < 14.4% or a simulated altitude >3,000 m) (Girard, Brocherie, & Millet, 2017). Heat stress (elevated muscle temperature) enhances isolated sprint performance (Girard, Brocherie, & Bishop, 2015). This may be due to increased anaerobic ATP turnover in fast‐twitch fibers (Gray, de Vito, Nimmo, Farina, & Ferguson, 2006), improved muscle fiber contraction velocity (Farina, Arendt‐Nielsen, & Graven‐Nielsen, 2005; Gray et al., 2006), and/or enhanced glycolytic enzyme activity (Febbraio et al., 1996; Stienen, Kiers, Bottinelli, & Reggiani, 1996). However, when core temperature is elevated above 38.5℃ (i.e., moderate hyperthermia), RSA is impaired due to increased central fatigue development (Drust, Rasmussen, Mohr, Nielsen, & Nybo, 2005).

The independent effects of hypoxia and heat stress on performance and physiological responses during repeated‐sprint exercise are well described. Girard, Brocherie, Morin, and Millet (2016) compared acute performance, physiological, and mechanical alterations during repeated running sprint exercise between severely hot (38℃) and hypoxic (FiO_2_: 13.3% [a simulated altitude of 3,600 m]) environments. They found that hot environment with moderate hyperthermia (~38.5℃) attenuated sprint performance in the initial exercise stage than hypoxic environment, whereas a larger performance decrement was observed under hypoxic versus heat stress. However, combined effects of hot and hypoxic environments on repeated‐sprint performance remain unknown. To date, hypoxia and heat stress when combined negatively affect performance of continuous, prolonged exercises compared to each stressor alone, as a result of the combined effect of reduced oxygen availability and increased core temperature (Aldous et al., 2015; Girard & Racinais, 2014). It remains possible that adding heat exposure during a repeated‐sprint exercise (maximal efforts during an exercise of rather short duration) in hypoxia has positive effects on repeated‐sprint performance and associated physiological responses.

The intention of this study was therefore to investigate the isolated and combined effects of heat stress and moderate hypoxia on repeated‐sprint performance and associated physiological responses. When hot and hypoxia are combined, we hypothesized that [1] the initial sprint performance would be improved, despite reduced systemic oxygen uptake, and that [2] the exercise‐induced increase in blood lactate level and acid‐base disturbance would be exacerbated.

## 2 MATERIALS AND METHODS

### 2.1 Subjects

Ten male college students (mean ± standard error [SE]; age, 19.6 ± 0.3 years; height, 173.3 ± 2.2 cm; weight, 71.6 ± 1.8 kg) volunteered to participate in the study. All subjects were highly trained athletes (training experience >5 years, competing at national and international levels) who underwent intensive training on 6 days/week (2 hr/day). They had not experienced specific training using hot and hypoxic environments for at least 6 months before the experiments. They were instructed to avoid intense exercise and caffeine and alcohol intake for 24 hr before each experimental trial. Subjects were informed about the purpose of the study, and the possible risks and benefits, and provided written informed consent. The study was approved by the Research Ethics Committee of Ritsumeikan University, Japan and conducted in accordance with Declaration of Helsinki (2013).

### 2.2 Experiment overview

All subjects visited the laboratory on five occasions during the experiment. The first visit was a familiarization session for the repeated‐sprint exercise, which consisted of two sets of 3 × 10‐s maximal sprints (40 s between sprints) under thermoneutral normoxic condition (20℃, 50% relative humidity [rH], FiO_2_: 20.9%). Visits 2–5 were the main experimental trials, which consisted of a repeated‐sprint exercise under four different conditions: control (CON; 20℃, 50% rH, FiO_2_: 20.9% [sea level]); hypoxia (HYP; 20℃, 50% rH, FiO_2_: 14.5% [a simulated altitude of 3,000 m]); hot (HOT; 35℃, 50% rH, FiO_2_: 20.9% [sea level]); and hot + hypoxia (HH; 35℃, 50% rH, FiO_2_: 14.5% [a simulated altitude of 3,000 m]). This study used a single‐blind, cross‐over design. Each trial was separated by at least 1 week, and the order of conditions was randomized and counterbalanced. All trials (including the familiarization session) were performed in a large (14.8 m^2^) normobaric hypoxic chamber (FCC‐5000S; Fuji Medical Science Co. Ltd.), which allowed continuous monitoring of the O_2_ and carbon dioxide (CO_2_) concentrations in the chamber. For the HYP and HH conditions, normobaric hypoxia was created by blowing nitrogen into the chamber.

### 2.3 Exercise protocol

**FIGURE 1:**
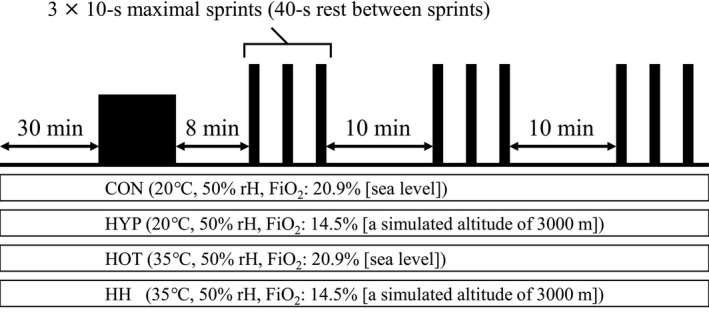
Protocol overview

On the experimental trial days, each subject arrived at the laboratory at the same time in the morning following overnight fasting. After baseline measurements in the thermoneutral normoxic environment (20℃, 50% rH, FiO_2_: 20.9%), the subjects entered the chamber and remained in a seated position for 30 min; then, they started the prescribed warm‐up exercise, consisting of a 5‐min period of submaximal cycling (60 rpm, 60 W) followed by three 3‐s maximal sprints. After 8 min of rest, the subjects performed the experimental exercise, which included three sets of 3 × 10‐s maximal sprints (a total of nine sprints). There was a 40‐s passive recovery period between sprints and a 10‐min passive rest period between sets (Figure 1). We selected this exercise protocol, consisting of relatively small number of sprints with long rest periods, to avoid marked reduction of power output during the latter stages of exercise. The exercise was performed on an electromagnetic braked cycle ergometer (Power Max VⅢ; Konami Corp.). The pedaling load was fixed at 7.5% of body weight (0.74 N kg^‐1^ of body weight). The subjects were allowed to consume water ad libitum throughout the exercise. Body weight was measured before and after all exercises and measured body weight was corrected by water volume consumed. On completion of the trial, the subjects exited the chamber and rested in a seated position for 60 min in a thermoneutral normoxic environment (20℃, 50% rH, FiO_2_: 20.9%).

### 2.4 Measurements

Peak and mean power outputs during each 10‐s sprint were measured under each condition. Total work of all sprints was calculated by the sum of mechanical work for nine sprints. The fatigue index (FI) over all sprints was calculated using the best mean power output (MP_best_) and the worst mean power output (MP_worst_) in watts (W) (Girard et al., 2011).

$$\text{FI}\left(\%\right)=100\times \left[{\text{MP}}_{\text{best}}\left(W\right)\;-\;{\text{MP}}_{\text{worst}}\left(W\right)\right]\;/{\text{MP}}_{\text{best}}\left(W\right)$$

The sprint decrement score (S_dec_) was also calculated using mean power output for each sprint (MP_1_‐MP_9_) and MP_best_ (Girard et al., 2011).

$$S_{\text{dec}}\left(\%\right)=100\times \left[1-\left({\text{MP}}_{1}\;+\;{\text{MP}}_{2}\;+\;{\text{MP}}_{3}\;+\;\ldots \;+\;{\text{MP}}_{9}\right)/\left(9\;\times \;{\text{MP}}_{\text{best}}\right)\right]$$

Muscle temperature was monitored noninvasively using the zero‐heat‐flow method with a surface thermometer (CM‐210; Terumo Corp., Tokyo, Japan) (Togwa, Nemoto, Yamazaki, & Kobayashi, 1976; Yamakage, Iwasaki, & Namiki, 2002). The probe was attached to the muscle belly of the *vastus lateralis* (middle of the thigh) of the left leg. Skin temperature was monitored using a data logger (N543; Nikkiso Therm Co. Ltd.) and wire probes (ITP082‐24; Nikkiso Therm Co. Ltd.) attached to the muscle belly on the left side of the chest, arm, thigh, and calf. The mean skin temperature was calculated according to Ramanathan (1964). Under each condition, muscle and skin temperatures were obtained every 2 s from the end of the 30‐min resting period (60 s of data) through competition of the exercise.

Oxygen uptake ( ${\dot{V}O}_{2}$ ), carbon dioxide production ( ${\dot{V}\text{CO}}_{2}$ ), and minute ventilation ( $\dot{V}E$ ) were measured breath‐by‐breath during both exercise (10 s) and rest (40 s) periods with an automatic gas analyzer (AE‐300S; Minato Medical Science Co. Ltd., Tokyo, Japan). Cardiorespiratory data were subsequently averaged every 5 s. Heart rate (HR) and arterial oxygen saturation (SpO_2_) were continuously monitored every second during the exercise using a wireless HR monitor (RCX5; Polar Electro) and a finger pulse oximeter on the right forefinger (PULSOX‐Me300; Teijin Pharma Ltd., Tokyo, Japan), respectively. The ${\dot{V}O}_{2}$ , ${\dot{V}\text{CO}}_{2}$ , $\dot{V}E$ , HR, and SpO_2_ are expressed as mean values for each exercise set, which included three 10‐s sprints and two 40‐s rest period between sprints.

Blood samples were collected before the subjects entered the chamber (baseline), and 0, 3, 5, 30, and 60 min after exercise via a canula inserted into the antecubital vein. Capillary blood samples were collected from a fingertip immediately after the first and second sets of exercise. Blood glucose and lactate concentrations were measured immediately after blood collection using a glucose analyzer (FreeStyle Freedom Lite; Nipro Corp.) and a lactate analyzer (Lactate Pro 2; Arkray Inc.), respectively. Blood pH, partial pressure of oxygen (PO_2_), partial pressure of carbon dioxide (PCO_2_), base excess (BE), and bicarbonate (HCO_3_^‐^) levels were determined immediately after blood collection using a blood gas analyzer (OPTI CCA‐TS2; OPTI Medical Systems Inc.). Hemoglobin and hematocrit values were obtained to calculate plasma volume change (ΔPV) using an estimative equation (Dill & Costill, 1974). Absolute concentrations of the blood variables were corrected using ΔPV to eliminate the influence of exercise‐induced hemoconcentration. Plasma samples were obtained by centrifugation for 10 min at 4℃ and stored at −80℃ for subsequent analysis conducted after completion of all testing sessions of all participants. Plasma adrenaline, noradrenaline, and glucagon concentrations were measured in a clinical laboratory before and immediately after exercise (SRL Inc., Tokyo, Japan). The coefficient of variation was 6.6% for adrenaline, 6.5% for noradrenaline, and 9.0% for glucagon.

Ratings of perceived difficulty breathing (RPE_breath_) and lower limb discomfort (RPE_leg_) were assessed immediately after each set using a 10‐point scale (Christian, Bishop, Billaut, & Girard, 2014). Thermal sensation was measured using a 9‐point scale (1 = “very cold”, 9 = “very hot”) (Zhang, Arens, Huizenga, & Han, 2010).

### 2.5 Statistical analysis

All statistical analyses were performed using SPSS statistical software (IBM Corp.). All data are expressed as means ± SE, and 95% confidence intervals (CI) are also presented.

A two‐way repeated‐measures analyses of variance (ANOVA) was used to assess the interaction and main effects of condition and time. For the average of power output, cardiorespiratory variables, and perceptual responses, one‐way repeated‐measures ANOVA was conducted to evaluate the main effect of condition. Significant interactions and main effects were further analyzed using the Tukey–Kramer post hoc. *p*‐values < .05 were deemed to indicate statistical significance.

## 3 RESULTS

### 3.1 Power output

**FIGURE 2:**
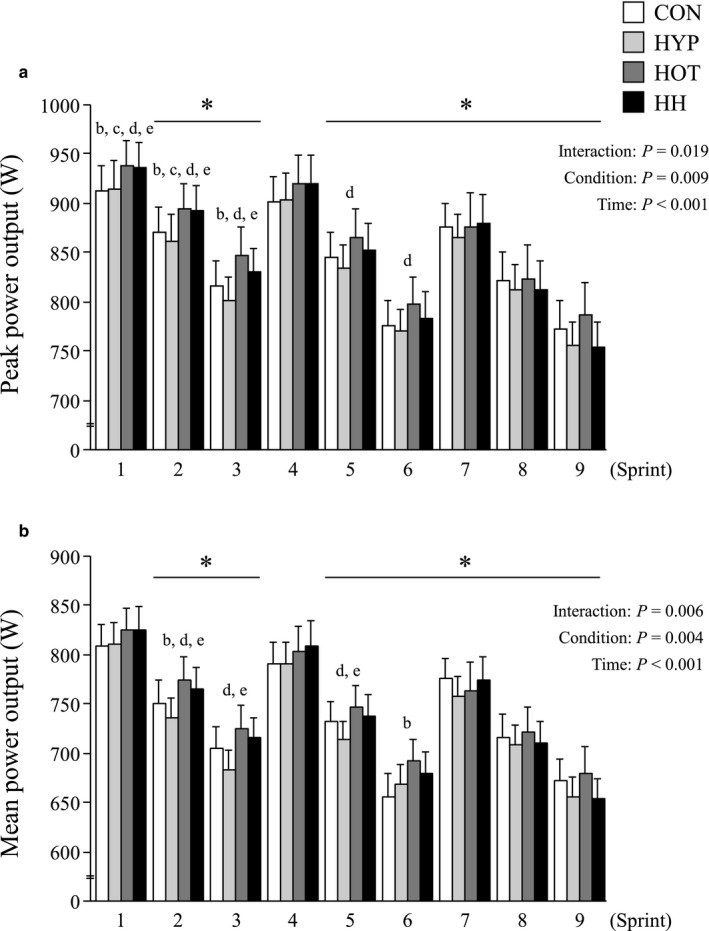
Changes in peak (a) and mean power outputs (b) during exercise. Values are means ± SE. *: *p* < .05 vs. sprint 1, b: *p* < .05 CON vs. HOT, c: *p* < .05 CON vs. HH, d: *p* < .05 HYP vs. HOT, e: *p* < .05 HYP vs. HH

The peak power outputs in HOT and HH were significantly greater than those in CON and HYP during the first and second sprints (*p* < .05), but not during any subsequent sprints (Figure 2). Compared to HYP, mean power output was elevated in both HOT and HH for sprints number 2, 3, and 5 (*p* < .05; Figure 2). There was no significant difference between CON and HYP for both peak and mean power outputs. Average of mean power output for sprint 1–9 was significantly higher in HOT (748 ± 23 W [CI: 703–793 W]) than HYP (725 ± 20 W [CI: 686–763 W], *p* < .05), and total work produced throughout nine sprints was also higher in HOT (67.3 ± 2.1 kJ [CI: 63.3–71.3 kJ]) than in HYP (65.2 ± 1.8 kJ [CI: 61.8–68.7 kJ], *p* < .05). However, FI and S_dec_ did not differ significantly between conditions (averaged values for four conditions; FI: 18.5 ± 0.9% [CI: 16.7%–20.3%], S_dec_: 9.7 ± 0.6% [CI: 8.6%–10.9%]).

### 3.2 Body temperature

**FIGURE 3:**
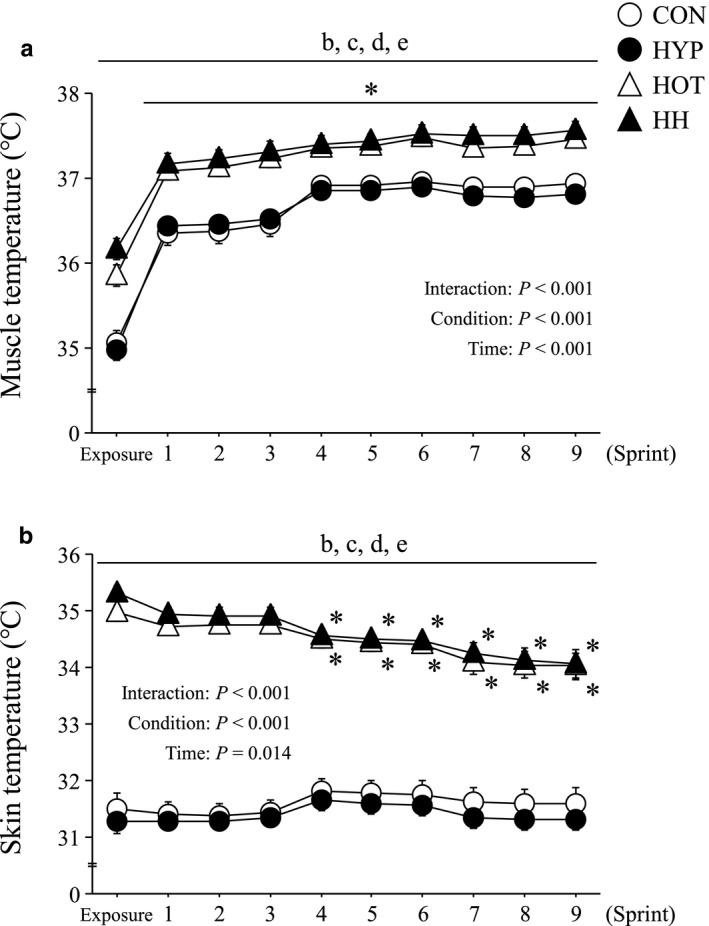
Changes in muscle (a) and skin temperatures (b) during exercise. Values are means ± SE. *: *p* < .05 vs. sprint 1, b: *p* < .05 CON vs. HOT, c: *p* < .05 CON vs. HH, d: *p* < .05 HYP vs. HOT, e: *p* < .05 HYP vs. HH

Both muscle and skin temperatures were significantly higher during both HOT and HH than CON and HYP at all time points (*p* < .05; Figure 3).

### 3.3 Cardiorespiratory variables

The VO_2_ was significantly lower during both HYP and HH than CON and HOT, for all exercise sets (*p* < .05; Table 1). Furthermore, the mean VO_2_ was significantly higher in HOT compared to any other condition (*p* < .05). The mean VE was significantly higher in HYP than in CON, and significantly higher in HH than in CON and HOT (*p* < .05; Table 1).

**TABLE 1:** Cardiorespiratory variables during each set of exercise

	Set 1	Set 2	Set 3	Average	ANOVA		
					Interaction	Condition	Time
VO_2_ (mL·min^−1^)					0.507	<0.001	0.134
CON	2,266 ± 66 (2137–2395)	2,301 ± 60 (2183–2418)	2,260 ± 55 (2152–2368)	2,275 ± 59 (2160–2391)			
HYP	1901 ± 61^†^ (1781–2021)	1891 ± 58^†^ (1777–2005)	1,850 ± 64^†^ (1724–1975)	1881 ± 59^†^ (1766–1996)			
HOT	2,422 ± 70^†^, ^‡^ (2284–2559)	2,458 ± 50^†^, ^‡^ (2360–2557)	2,417 ± 62^†^, ^‡^ (2296–2539)	2,432 ± 57^†^, ^‡^ (2321–2544)			
HH	2029 ± 76^†^, ^‡^, ^§^(1881–2178)	2050 ± 75^†^, ^‡^, ^§^ (1903–2197)	1959 ± 74^†^, ^§^ (1813–2105)	2013 ± 72^†^, ^‡^, ^§^ (1873–2153)			
VCO_2_ (mL·min^−1^)					0.111	0.001	<0.001
CON	2,666 ± 78 (2512–2819)	2,302 ± 68* (2169–2436)	2,117 ± 60* (1999–2234)	2,361 ± 59 (2246–2477)			
HYP	2,749 ± 78 (2597–2902)	2,256 ± 68* (2122–2389)	2028 ± 84* (1863–2193)	2,344 ± 67 (2212–2477)			
HOT	2,878 ± 86^†^ (2709–3048)	2,497 ± 74*, †, ‡ (2352–2642)	2,262 ± 76^*‡^ (2112–2412)	2,546 ± 65^†^, ^‡^ (2418–2674)			
HH	2,849 ± 106 (2642–3056)	2,482 ± 74*, †, ‡ (2337–2627)	2,152 ± 75* (2004–2299)	2,494 ± 71^‡^ (2354–2635)			
VE (L·min^−1^)					0.070	<0.001	0.064
CON	95.9 ± 4.5 (87.0–104.8)	104.8 ± 5.4 (94.2–115.4)	105.9 ± 6.5 (93.2–118.6)	102.2 ± 5.2 (92.1–112.3)			
HYP	106.0 ± 5.7^†^ (94.9–117.1)	112.6 ± 5.9^†^ (101.0–124.2)	109.7 ± 8.2 (93.6–125.9)	109.4 ± 6.2^†^ (97.2–121.7)			
HOT	98.3 ± 3.9 (90.7–105.8)	107.5 ± 4.6 (98.5–116.5)	107.0 ± 6.4 (94.5–119.6)	104.3 ± 4.4 (95.7–112.8)			
HH	105.1 ± 5.7^†^ (94.0–116.2)	119.4 ± 6.7^†^, ^‡^, ^§^ (106.3–132.4)	112.4 ± 6.8 (99.1–125.6)	112.3 ± 6.0^†^, ^§^ (100.5–124.1)			
HR (bpm)					0.439	0.056	0.106
CON	147 ± 3 (141–153)	153 ± 3 (147–159)	154 ± 3 (147–161)	151 ± 3 (145–157)			
HYP	142 ± 10 (122–161)	153 ± 4 (145–160)	151 ± 4 (142–159)	148 ± 5 (139–158)			
HOT	154 ± 3 (147–160)	158 ± 2 (154–163)	152 ± 3 (146–159)	155 ± 2 (151–158)			
HH	156 ± 3 (149–163)	160 ± 4 (152–168)	157 ± 5 (147–167)	158 ± 4 (150–166)			
SpO_2_ (%)					0.321	< 0.001	0.361
CON	92.6 ± 1.2 (90.2–94.9)	90.6 ± 2.2 (86.3–94.8)	92.2 ± 4.3 (89.6–94.9)	91.8 ± 1.4 (89.1–94.4)			
HYP	85.0 ± 1.4^†^ (82.2–87.7)	86.1 ± 0.7^†^ (84.6–87.5)	86.3 ± 2.1^†^ (85.0–87.6)	85.8 ± 0.8^†^ (84.2–87.4)			
HOT	93.3 ± 1.0^‡^ (91.4–95.3)	92.6 ± 1.3^‡^ (90.0–95.1)	94.0 ± 1.5^‡^ (93.0–94.9)	93.3 ± 0.8^‡^ (91.7–94.9)			
HH	86.9 ± 0.7^†^, ^§^ (85.6–88.2)	85.6 ± 0.7^†^, ^§^ (84.3–87)	85.8 ± 2.0^†^, ^§^ (84.6–87.0)	86.1 ± 0.6^†^, ^§^ (85.0–87.3)			

Note:

Values are means ± SE (95% CI).

* *p* < .05 vs. set 1,

^†^ *p* < .05 vs. CON,

^‡^ *p* < .05 vs. HYP,

^§^ *p* < .05 vs. HOT.

HR did not differ significantly among conditions, although the mean value tended to be higher under the HH (*p* = .056). The SpO_2_ was significantly lower in HYP and HH compared with CON and HOT for all sets and average value over the nine sprints (*p* < .05; Table 1).

### 3.4 Blood variables

**FIGURE 4:**
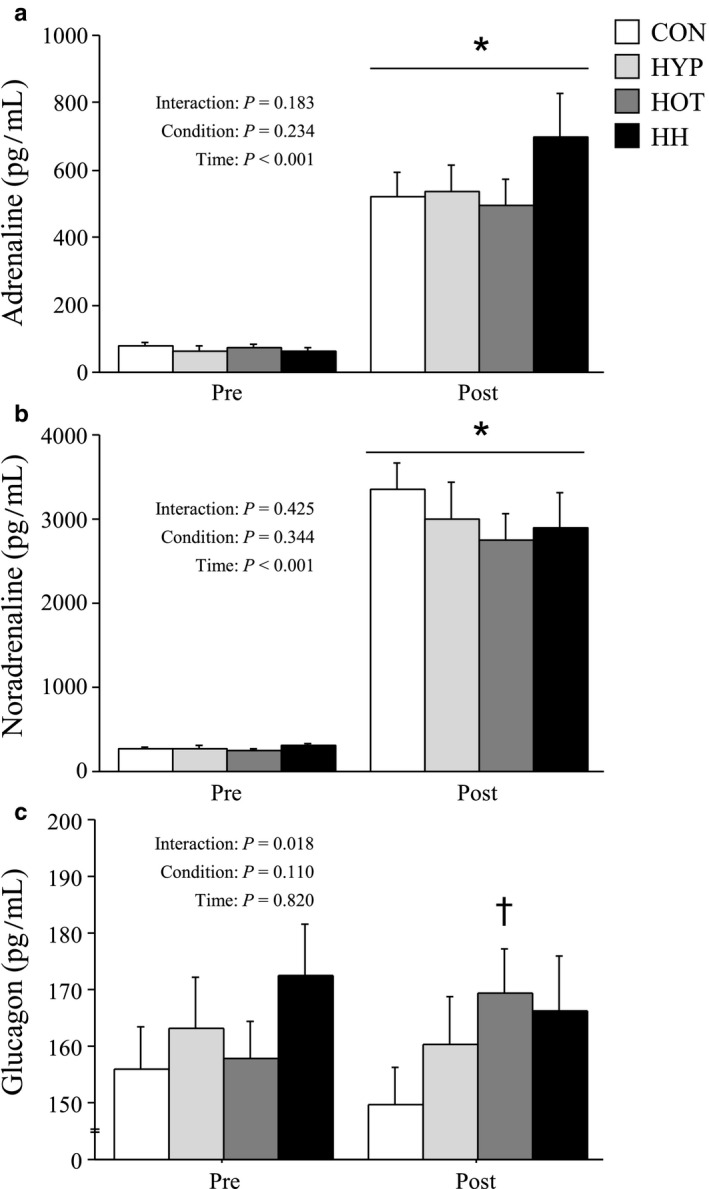
Plasma adrenaline (a), noradrenaline (b), and glucagon concentrations (c) before and after exercise. Values are means ± SE. *: *p* < .05 vs. Pre, †: *p* < .05 vs. CON

Both plasma adrenaline and noradrenaline concentrations were significantly elevated after the exercise, but there was no significant difference among four conditions (Figure 4). Plasma glucagon concentration slightly increased from pre‐ to postexercise in HOT, and values measured after exercise were significantly higher in HOT than in CON (*p* < .05; Figure 4).

Blood lactate and glucose concentrations were significantly increased after the exercise (*p* < .05), although no significant difference was observed among conditions (Table 2). Blood PO_2_ did not differ significantly among conditions; however, blood PCO_2_ was significantly lower in HH compared to both CON and HYP after set 3 (*p* < .05; Table 2). We found no significant differences in blood pH, BE, HCO_3_^‐^, and ΔPV among conditions (Table 3). Furthermore, change in body weight after exercise did not differ significantly among conditions (CON: 0.05 ± 0.05 kg [CI: −0.05 to 0.15 kg], HYP: 0.03 ± 0.05 kg [CI: −0.07 to 0.13 kg], HOT: −0.20 ± 0.06 kg [CI: −0.32 to −0.08 kg], HH: −0.13 ± 0.17 kg [CI: −0.46 to 0.20 kg]).

**TABLE 2:** Blood lactate, glucose concentrations and blood gas variables before and after exercise

	Baseline	Set 1	Set 2	Set 3	Post‐exercise				ANOVA		
					3 min	5 min	30 min	60 min	Interaction	Condition	Time
Lactate (mmol·L^−1^)									0.858	0.643	<0.001
CON	1.1 ± 0.1 (0.9–1.3)	10.2 ± 1.0* (8.2–12.2)	15.5 ± 1.5* (12.6–18.4)	17.1 ± 1.2* (14.7–19.5)	16.1 ± 1.0* (14.1–18.1)	16.3 ± 1.0* (14.3–18.3)	9.9 ± 0.9* (8.1–11.7)	4.9 ± 0.5* (3.9–5.9)			
HYP	1.3 ± 0.1 (1.1–1.5)	11.0 ± 1.1* (8.8–13.2)	15.6 ± 0.8* (14.0–17.2)	17.3 ± 1.1* (15.1–19.5)	15.7 ± 0.9* (13.9–17.5)	16.1 ± 0.9* (14.3–17.9)	10.4 ± 0.7* (9.0–11.8)	5.2 ± 0.7* (3.8–6.6)			
HOT	1.3 ± 0.1 (1.1–1.5)	10.9 ± 0.6* (9.7–12.1)	14.2 ± 1.0* (12.2–16.2)	16.5 ± 1.0* (14.5–18.5)	15.9 ± 0.9* (14.1–17.7)	16.3 ± 0.8* (14.7–17.9)	9.2 ± 0.9* (7.6–10.8)	4.3 ± 0.4* (3.5–5.1)			
HH	1.2 ± 0.1 (1.0–1.4)	11.3 ± 0.6* (10.1–12.5)	15.3 ± 0.6* (14.1–16.5)	17.2 ± 1.1* (15.0–19.4)	16.5 ± 0.8* (14.9–18.1)	15.8 ± 0.7* (14.4–17.2)	10.4 ± 0.7* (9.0–11.8)	4.7 ± 0.5* (3.7–5.7)			
Glucose (mg·dL^−1^)									0.092	0.247	<0.001
CON	83 ± 2 (78–87)	93 ± 3* (87–99)	96 ± 4* (88–103)	93 ± 3* (86–99)	101 ± 4* (93–108)	101 ± 5* (91–111)	89 ± 4 (81–97)	75 ± 3 (69–81)			
HYP	88 ± 2 (84–92)	88 ± 3 (82–93)	94 ± 3 (88–101)	97 ± 6 (86–108)	100 ± 7* (86–114)	104 ± 7 (90–117)	93 ± 6 (82–105)	80 ± 3 (75–85)			
HOT	86 ± 2 (82–90)	97 ± 2* (92–102)	98 ± 3* (92–103)	95 ± 3 (88–102)	100 ± 4* (92–107)	101 ± 4* (93–109)	91 ± 5 (81–101)	84 ± 3 (77–90)			
HH	86 ± 3 (81–92)	95 ± 2 (90–99)	97 ± 3 (91–103)	98 ± 4 (91–106)	109 ± 6* (98–120)	108 ± 5* (99–117)	99 ± 7* (87–112)	87 ± 5 (78–96)			
PO_2_ (kPa)									0.164	0.200	<0.001
CON	8.85 ± 0.64 (7.60–10.10)			5.18 ± 0.84* (3.53–6.83)	11.75 ± 0.40* (10.97–12.53)	13.11 ± 0.48* (12.17–14.05)	10.49 ± 0.52 (9.47–11.51)	8.35 ± 0.62 (7.13–9.57)			
HYP	8.51 ± 0.64 (7.26–9.76)			3.98 ± 0.51* (2.98–4.98)	10.94 ± 0.59* (9.78–12.1)	12.02 ± 0.50* (11.04–13.00)	9.09 ± 0.69 (7.74–10.44)	7.47 ± 0.77 (5.96–8.98)			
HOT	8.30 ± 0.56 (7.20–9.40)			6.41 ± 0.73 (4.98–7.84)	10.17 ± 0.46 (9.27–11.07)	12.06 ± 0.56* (10.96–13.16)	10.22 ± 0.67* (8.91–11.53)	8.16 ± 0.81 (6.57–9.75)			
HH	8.74 ± 1.02 (6.74–10.74)			6.05 ± 0.67* (4.74–7.36)	11.52 ± 0.42* (10.70–12.34)	11.81 ± 0.39* (11.05–12.57)	10.39 ± 0.36 (9.68–11.10)	8.37 ± 0.67 (7.06–9.68)			
PCO_2_ (kPa)									<0.001	0.044	<0.001
CON	5.64 ± 0.14 (5.37–5.91)			6.09 ± 0.49 (5.13–7.05)	4.07 ± 0.23* (3.62–4.52)	3.84 ± 0.16* (3.53–4.15)	4.28 ± 0.11* (4.06–4.50)	5.14 ± 0.17 (4.81–5.47)			
HYP	5.70 ± 0.11 (5.48–5.92)			6.02 ± 0.33 (5.37–6.67)	4.08 ± 0.18* (3.73–4.43)	4.02 ± 0.10* (3.82–4.22)	4.37 ± 0.16* (4.06–4.68)	5.20 ± 0.17 (4.87–5.53)			
HOT	5.71 ± 0.19 (5.34–6.08)			5.19 ± 0.36 (4.48–5.90)	4.15 ± 0.23* (3.70–4.60)	3.91 ± 0.18* (3.56–4.26)	4.29 ± 0.08* (4.13–4.45)	5.20 ± 0.12 (4.96–5.44)			
HH	5.98 ± 0.21 (5.57–6.39)			4.51 ± 0.29*, †, ‡ (3.94–5.08)	3.82 ± 0.10* (3.62–4.02)	3.84 ± 0.12* (3.60–4.08)	4.13 ± 0.11* (3.91–4.35)	5.12 ± 0.15* (4.83–5.41)			

Note:

Values are means ± SE (95% CI).

* *p* < .05 vs. Baseline,

^†^ *p* < .05 vs. CON,

^‡^ *p* < .05 vs. HYP.

**TABLE 3:** Acid‐base balance and plasma volume shift before and after exercise

	Baseline	Set 3	Post‐exercise				ANOVA		
			3 min	5 min	30 min	60 min	Interaction	Condition	Time
pH							0.818	0.016	<0.001
CON	7.42 ± 0.005 (7.41–7.42)	7.22 ± 0.036* (7.14–7.29)	7.19 ± 0.014* (7.16–7.22)	7.19 ± 0.013* (7.16–7.21)	7.33 ± 0.016* (7.30–7.36)	7.38 ± 0.011 (7.36–7.40)			
HYP	7.41 ± 0.005 (7.40–7.42)	7.20 ± 0.016* (7.17–7.23)	7.19 ± 0.015* (7.16–7.22)	7.19 ± 0.017* (7.16–7.23)	7.33 ± 0.012* (7.31–7.36)	7.39 ± 0.009 (7.37–7.41)			
HOT	7.41 ± 0.004 (7.40–7.41)	7.21 ± 0.015* (7.18–7.24)	7.21 ± 0.009* (7.19–7.23)	7.21 ± 0.010* (7.19–7.23)	7.35 ± 0.012* (7.32–7.37)	7.39 ± 0.006 (7.38–7.40)			
HH	7.41 ± 0.010 (7.39–7.43)	7.24 ± 0.012* (7.22–7.27)	7.22 ± 0.014* (7.19–7.25)	7.22 ± 0.013* (7.19–7.24)	7.36 ± 0.010* (7.34–7.38)	7.40 ± 0.010 (7.38–7.42)			
BE (mmol·L^−1^)							0.014	0.495	<0.001
CON	1.7 ± 0.5 (0.7 ~ 2.7)	−9.4 ± 0.7* (−10.7~−8.1)	−12.5 ± 0.4* (−13.3~−11.7)	−13.1 ± 0.5* (−14.1~−12.1)	−7.5 ± 0.7* (−8.8~−6.2)	−2.2 ± 0.6* (−3.4~−1.0)			
HYP	1.5 ± 0.4 (0.7 ~ 2.3)	−8.6 ± 0.8* (−10.1~−7.0)	−12.5 ± 0.6* (−13.7~−11.4)	−12.7 ± 0.6* (−13.9~−11.4)	−7.1 ± 0.7* (−8.5~−5.6)	−1.5 ± 0.5* (−2.6~−0.5)			
HOT	1.3 ± 0.6 (0.1 ~ 2.5)	−9.2 ± 0.4* (−10.1~−8.4)	−11.5 ± 0.4* (−12.2~−10.7)	−12.3 ± 0.4* (−13.2~−11.5)	−6.8 ± 0.5* (−7.8~−5.8)	−1.8 ± 0.3* (−2.4~−1.2)			
HH	3.0 ± 0.3 (2.4 ~ 3.6)	−9.2 ± 0.9* (−10.9~−7.5)	−12.2 ± 0.7* (−13.5~−11.0)	−12.4 ± 0.7* (−13.8~−11.1)	−6.5 ± 0.7* (−8.0~−5.1)	−1.0 ± 0.6* (−2.1 ~ 0.0)			
HCO_3_^‐^ (mmol·L^−1^)							<0.001	0.561	<0.001
CON	26.5 ± 0.6 (25.3–27.7)	13.0 ± 0.9* (11.3–14.7)	9.1 ± 0.4* (8.3–9.9)	8.8 ± 0.4* (7.9–9.6)	15.0 ± 0.7* (13.7–16.3)	21.2 ± 0.8* (19.6–22.9)			
HYP	26.5 ± 0.5 (25.5–27.5)	14.0 ± 1.1* (11.9–16.0)	9.6 ± 0.8* (8.1–11.1)	9.5 ± 0.7* (8.2–10.8)	15.7 ± 0.9* (14.0–17.4)	21.4 ± 0.9* (19.7–23.2)			
HOT	26.3 ± 0.8 (24.7–27.9)	12.0 ± 0.7* (10.6–13.4)	9.9 ± 0.7* (8.6–11.2)	9.6 ± 0.5* (8.6–10.5)	16.2 ± 0.6* (14.9–17.4)	22.5 ± 0.7* (21.1–23.8)			
HH	28.0 ± 0.5 (27.0–29.0)	11.5 ± 1.2* (9.2–13.8)	9.7 ± 0.5* (8.7–10.7)	9.8 ± 0.5* (8.8–10.7)	15.8 ± 0.7* (14.5–17.0)	22.7 ± 0.7* (21.3–24.1)			
ΔPV (%)							0.031	0.606	<0.001
CON	0.0 ± 0.0 (0.0–0.0)	−20.8 ± 1.4* (−23.5~−18.1)	−19.6 ± 1.2* (−22.0~−17.3)	−18.1 ± 1.7* (−21.4~−14.8)	−8.9 ± 1.8* (−12.4~−5.4)	−5.2 ± 1.4* (−8.0~−2.4)			
HYP	0.0 ± 0.0 (0.0–0.0)	−19.3 ± 1.4* (−22.1~−16.6)	−17.5 ± 1.8* (−21.1~−14.0)	−17.1 ± 2.1* (−21.2~−13.1)	−8.0 ± 1.5* (−10.9~−5.1)	−7.2 ± 2.2* (−11.6~−2.8)			
HOT	0.0 ± 0.0 (0.0–0.0)	−21.7 ± 0.8* (−23.3~−20.0)	−19.4 ± 1.4* (−22.1~−16.7)	−16.9 ± 1.6* (−20.1~−13.7)	−6.1 ± 2.1* (−10.2~−2.0)	−1.8 ± 2.1 (−5.8 ~ 2.3)			
HH	0.0 ± 0.0 (0.0–0.0)	−20.8 ± 2.4* (−25.6~−16.0)	−16.1 ± 1.7* (−19.4~−12.7)	−15.1 ± 2.1* (−19.1~−11.0)	−7.7 ± 2.2* (−12.0~−3.3)	−2.8 ± 1.6 (−5.9 ~ 0.3)			

Note:

Values are means ± SE (95% CI).

* *p* < .05 vs. Baseline.

### 3.5 Perceptual responses

Compared to CON, higher RPE_breath_ (CON: 6.5 ± 0.5 [CI: 5.5–7.5]; HYP: 7.5 ± 0.4 [CI: 6.7–8.2]; HOT: 7.6 ± 0.3 [CI: 7.0–8.2]; HH: 7.8 ± 0.3 [CI: 7.1–8.4]) and RPE_leg_ (CON: 7.3 ± 0.5 [CI: 6.4–8.2]; HYP: 8.1 ± 0.5 [CI: 7.2–9.1]; HOT: 8.0 ± 0.4 [CI: 7.2–8.7]; HH: 7.8 ± 0.4 [CI: 7.1–8.6]) values were measured for HYP, HOT, and HH (*p* < .05). Thermal sensation was significantly elevated with heat exposure (HOT: 8.2 ± 0.1 [CI: 8.0–8.5] and HH: 7.8 ± 0.4 [CI: 7.0–8.7] versus CON: 5.7 ± 0.2 [CI: 5.2–6.1]; HYP: 5.3 ± 0.4 [CI: 4.5–6.2], *p* < .05).

## 4 DISCUSSION

We investigated performance, energy metabolism, acid–base balance, and endocrine responses during repeated‐sprint exercise under combined hot and hypoxic conditions compared to each stressor alone. Our main finding was that both peak and mean power output during the first set of exercise were significantly improved with heat exposure (higher in both HOT and HH than both CON and HYP), while VO_2_ and SpO_2_ levels were lower in HYP and HH, and body temperatures were higher in HOT and HH. However, exercise‐induced changes in the blood levels of lactate and glucose, plasma adrenaline and noradrenaline concentrations, and acid–base balance were not significantly different among the conditions. The addition of heat stress when sprinting repeatedly in hypoxia enhances repeated‐sprint performance with limited O_2_ availability.

### 4.1 Performance outcomes

We expected that an elevated muscle temperature would increase power output during the initial phase of the exercise under the HOT and HH conditions. Previous studies found that heat stress (increased muscle temperature) increased power output during a single sprint (Ball, Burrows, & Sargeant, 1999; Girard, Bishop, & Racinais, 2013; Linnane, Bracken, Brooks, Cox, & Ball, 2004). Possible explanations include an increased anaerobic ATP turnover in fast‐twitch fibers (Gray et al., 2006), improved muscle fiber contraction velocity (Farina et al., 2005; Gray et al., 2006), and/or enhanced glycolytic enzyme activity (Febbraio et al., 1996; Stienen et al., 1996). In our study, average peak power output during the first exercise set (three sprints) was 3.0% and 2.3% higher under the HOT and HH conditions, respectively, compared with the CON condition. The magnitude of performance improvement in our study was comparable to that of a previous study, which reported 3.1% higher power output during completion of 10 × 6‐s repeated cycle sprint exercise in a hot (35℃) compared with thermoneutral (24℃) environment (Girard et al., 2013). However, another previous research found that hot environment (38℃) reduced initial sprint performance during repeated running sprint exercise (Girard et al., 2016). The inconsistent results may depend on differences in exercise modality, protocol design, and/or severity of heat stress between studies.

In the present study, the beneficial effect of the hot environment on power output was diminished during later phases of the exercise. As a plausible reason for this phenomenon, increased muscle temperature primarily improves anaerobic power output (Febbraio et al., 1996; Gray et al., 2006). Anaerobic energy contribution markedly decreases during the later phase of repeated‐sprint exercise, although it predominates during the initial sprint (Gaitanos, Williams, Boobis, & Brooks, 1993). However, considering specific exercise protocol with inserting long rest period every three sprints, other possibilities might be presumed.

Despite an increase in power output during the initial phase of exercise, the FI and S_dec_ did not differ across the four conditions in our study. Previous studies of exercise under combined hot and hypoxic conditions found either a reduction in the time to exhaustion during continuous cycling exercise (Girard & Racinais, 2014) or a decrease in the total and sprint distance covered during a simulated soccer protocol (Aldous et al., 2015) compared to each stressor alone. Acute hypoxia and heat stress have been shown to independently decrease endurance capacity (Nybo, Rasmussen, & Sawka, 2014; Rusko, Tikkanen, & Peltonen, 2004), whereas elevated core temperature (>38.5℃) impairs repeated‐sprint performance (Drust et al., 2005). Given that sprint performance was improved in HOT and HH in our study, these conditions unlikely caused severe hyperthermia at least during the first set of exercise. It was in line with a previous research which reported that hot environment (35℃) improved repeated‐sprint performance compared to thermoneutral environment (24℃) without severe hyperthermia (Girard et al., 2013). Furthermore, the ΔPV and decrease in body weight after exercise were similar among conditions, suggesting that the HOT and HH conditions did not cause dehydration. Therefore, the HH condition had positive (rather than negative) effects on repeated‐sprint performance.

### 4.2 Energy metabolism

Aerobic metabolism (VO_2_) and phosphocreatine resynthesis between sprints influence RSA (Bishop et al., 2011; Girard et al., 2011; Spencer, Bishop, Dawson, & Goodman, 2005). In the present study, averaged VO_2_ and SpO_2_ levels were lower in both HYP and HH compared with CON and HOT, suggesting that the aerobic energy supply was impaired (Ogura et al., 2006). However, the power output did not differ significantly between either CON and HYP conditions or HOT and HH conditions. These findings suggest that the anaerobic energy supply was augmented under both the HYP and HH conditions, which is consistent with previous observations (Ogawa et al., 2007; Ogura et al., 2006). Other studies found higher blood lactate concentrations after maximal sprint exercise under hypoxic (Bowtell et al., 2014) and hot (Linnane et al., 2004) conditions. In contrast, we did not observe significant differences in postexercise blood lactate levels or blood pH among conditions. Our exercise protocol (short‐duration exercise with relatively long rest periods between sessions) may explain discrepant findings between the present study and previous literatures. Furthermore, we measured lactate concentration in the blood only. Assessment of muscle lactate and glycogen levels, as well as lactate clearance, may bring further insights into adjustments in energy metabolism during repeated‐sprint exercise performed under combined hot and hypoxic conditions.

### 4.3 Acid–base balance

Exercise‐induced acid–base disturbances (e.g., changes in blood pH, BE, and HCO_3_^‐^) did not differ significantly among the conditions. Exercise under hot or hypoxic conditions causes protons (H^+^) to accumulate in the muscles (Hogan, Richardson, & Haseler, 1999; Sawka, Leon, Montain, & Sonna, 2011), leading to metabolic acidosis due to augmented anaerobic glycolysis. Furthermore, increased H^+^ may cause hyperventilation and subsequent respiratory alkalosis (Boedtkjer, 2018; Hamm, Nakhoul, & Hering‐Smith, 2015). We found that increases in VE and VCO_2_ during exercise under the HH condition were accompanied by lower PCO_2_ after exercise, indicating a degree of hypoxia‐induced (Swenson, 2016) and heat‐induced hyperventilation (Tsuji, Hayashi, Kondo, & Nishiyasu, 2016). However, the blood pH level did not differ significantly among the four conditions. There may be limited effects of adding hypoxia or heat stress on blood acid‐base balance, although repeated‐sprint exercise per se markedly decreased blood pH level even in CON.

### 4.4 Endocrine responses

The postexercise plasma adrenaline concentration was slightly higher under the HH condition than under the other three conditions, although the difference was not statistically significant. Catecholamines promote glycogenolysis in the muscle and liver, stimulating glucose mobilization to the blood (Zouhal, Jacob, Delamarche, & Gratas‐Delamarche, 2008). In this study, the HR and postexercise glucose concentrations were highest under the HH condition, which may have been due to higher plasma adrenaline levels. Previous studies have shown that exercise at submaximal intensity under hypoxic (an altitude of 1800 m) (Niess et al., 2003) and hot (40℃, 30% rH) conditions (Brenner, Zamecnik, Shek, & Shephard, 1997) augmented plasma catecholamine concentrations compared with the same exercise performed under normoxic/thermoneutral conditions. However, given that exercise intensity strongly affects sympathetic nerve activity (Zouhal et al., 2008), it may be that repeated all‐out sprint exercise itself maximizes catecholamine secretion under normoxic/thermoneutral conditions.

The plasma glucagon concentration did not change significantly immediately after exercise. Endurance exercise (> 20 min) generally increases the plasma glucagon concentration (Trefts, Williams, & Wasserman, 2015). Furthermore, an exercise‐induced increase in glucagon levels is an indicator of liver glycogenolysis and glucose production (Lavoie, Ducros, Bourque, Langelier, & Chiasson, 1997; Wasserman et al., 1989). However, previous findings suggest that glucose production in the liver is controlled by catecholamines rather than glucagon during high‐intensity exercise (Trefts et al., 2015). Therefore, the impact of glucagon on glucose regulation during repeated‐sprint exercise may be limited.

## 5 LIMITATION

The exercise protocol in the present study (relatively small number of sprints in each set interspersed with long rest periods) did not exactly match latest recommendations for typical repeated‐sprint training modalities (Brocherie et al., 2017). In addition, there was a large inter‐individual variability for responses to environmental stresses, although we selected “moderate” hot and hypoxic environments. Hence, SpO_2_ variability in healthy individuals increases substantially with the severity of simulated graded normobaric hypoxia for a wide range (12%–21%) of FiO_2_ values (Costello et al., 2020) Furthermore, we did not monitor core temperature to evaluate the severity of heat stress during exercise. Therefore, caution is required when interpreting the present results. Future studies should directly compare different exercise protocols, recruit a larger number of subjects, and monitor core temperature.

## 6 CONCLUSIONS

Repeated‐sprint exercise under combined hot and hypoxic condition induced significantly higher peak and mean power output compared with the control and hypoxia‐only conditions, despite reduced VO_2_ and SpO_2_ levels. These findings suggest that additional heat stress to hypoxia enhances repeated‐sprint performance, while maintaining low arterial oxygen saturation.

## CONFLICT OF INTEREST

There is no conflict of interest.

## AUTHOR CONTRIBUTIONS

Keiichi Yamaguchi designed the study, conducted data collection, analysis, and drafted manuscript; Nobukazu Kasai, Nanako Hayashi, and Haruka Yatsutani assisted in conceiving the study design and data collection; Olivier Girard assisted in analysis and revised manuscript. Kazushige Goto conceived the study design, assisted analysis, and revised manuscript. All authors reviewed and approved manuscript.
